# Effect of Fixed and sEMG-Based Adaptive Shared Steering Control on Distracted Driver Behavior

**DOI:** 10.3390/s21227691

**Published:** 2021-11-19

**Authors:** Zheng Wang, Satoshi Suga, Edric John Cruz Nacpil, Bo Yang, Kimihiko Nakano

**Affiliations:** 1 Institute of Industrial Science, The University of Tokyo, Tokyo 153-8505, Japan; knakano@iis.u-tokyo.ac.jp; 2 Department of Mechatronics Engineering, Technical University of Darmstadt, 64289 Darmstadt, Germany; satosuga95@gmail.com; 3 Corpy & Co., Inc., Tokyo 113-0033, Japan; edric@corpy.co.jp

**Keywords:** driver–automation shared control, haptic guidance steering, adaptive automation design, surface electromyography, driver distraction

## Abstract

Driver distraction is a well-known cause for traffic collisions worldwide. Studies have indicated that shared steering control, which actively provides haptic guidance torque on the steering wheel, effectively improves the performance of distracted drivers. Recently, adaptive shared steering control based on the forearm muscle activity of the driver has been developed, although its effect on distracted driver behavior remains unclear. To this end, a high-fidelity driving simulator experiment was conducted involving 18 participants performing double lane change tasks. The experimental conditions comprised two driver states: attentive and distracted. Under each condition, evaluations were performed on three types of haptic guidance: none (manual), fixed authority, and adaptive authority based on feedback from the forearm surface electromyography of the driver. Evaluation results indicated that, for both attentive and distracted drivers, haptic guidance with adaptive authority yielded lower driver workload and reduced lane departure risk than manual driving and fixed authority. Moreover, there was a tendency for distracted drivers to reduce grip strength on the steering wheel to follow the haptic guidance with fixed authority, resulting in a relatively shorter double lane change duration.

## 1. Introduction

Steering a car necessitates continuous real-time visual information from the road ahead. Distractions that inhibit safe and timely driver responses to this information are detrimental to driving safety; thus, technology designed to monitor and assist distracted drivers is a crucial advancement [1,2]. Although fully autonomous driving is not likely to be realized in the near future, partial automation is becoming more readily available in the form of steering assistance systems and driver attention monitoring [3]. Shared steering control systems assist drivers with curve negotiation and lane changes by producing a proper haptic guidance torque on the steering wheel [4,5], particularly when a lack of attention to visual information results from driver distraction or fatigue [6,7]. Decreased performance due to the lack of visual information can be compensated for by haptic guidance [8,9].

Some attempts to design an adaptive shared steering control system have been conducted to improve driver–automation cooperation. One way to adjust the driver–automation control authority is to address vehicle–environment factors, including vehicle position, steering wheel angle, and yaw rate [10,11,12,13]. A haptic shared steering control has been designed with an adaptive level of authority based on time-to-line crossing, and driver-in-the-loop experiments demonstrate the effectiveness of the proposed system on decreasing conflict torques [10]. Shared steering control with adaptive authority based on driver input torque has been designed to achieve smooth authority transfer from automated driving to manual driving [11]. Based on the data from lateral offset and lateral velocity to the road center line, a human driver’s driving intention and the desired maneuver have been recognized and used for designing a shared steering control system to avoid obstacles [12]. To further improve shared steering control performance, driver-in-the-loop model has been included in the optimal controller design [14,15,16,17,18,19,20]. A time-varying assistance factor has been developed to modulate the haptic steering torque, which is designed from an integrated driver-in-the-loop vehicle model, and the effectiveness of proposed haptic shared control method on driver–automation conflict management has been presented by driving tests conducted with high-fidelity simulations [14]. A Takagi–Sugeno fuzzy control approach has been proposed to deal with the time-varying driver activity parameter and vehicle speed under multiple system constraints to improve driver–automation shared steering performance [17]. A predictive control framework that uses a model of driver-in-the-loop steering dynamics to optimize the torque intervention with respect to the neuromuscular response of the driver has been presented. Results show the effectiveness of the proposed system in avoiding hazardous situations under different driver behaviors with driver-in-the-loop experiments [15]. Regarding the driver-in-the-loop control, a driver model that describes the driver steering motion as a sequence of identifiable motion primitives has been proposed for the calculation of the optimal torque [16]. In addition, a time-varying driver-in-the-loop path tracking model has been developed for shared steering control, where the preview behavior of the modelled driver is designed to mimic real human drivers [19]. By assuming that drivers can learn and incorporate the controller strategy into their internal model for predictive path following, a driver model has been proposed to include interactive steering behavior based on model predictive control for indirect shared control has been proposed [20].

Compared to adjusting the authority based on driver physiological status, the advantage of addressing vehicle–environment factors are apparent, as drivers do not need to wear bio-signal measuring equipment and driving performance data can be more easily recorded and processed in real time. However, the disadvantage is that it is difficult to establish an accurate mapping between driving performance data and individual driver behavior when compared with directly monitoring physiological status [21]. Moreover, it is sometimes not possible to establish an accurate driver-in-the-loop model for the controller design. An adaptive system has been designed to increase the automation authority, as driver control authority decreases owing to increased workload or decreased workload capacity [22], and this approach is preferable to an assistance system with fixed and rigid authority [23,24,25]. Driver workload can be estimated through physiological signal measurements such as eye and head movements [25] and surface electromyography (sEMG) signals [7]. For steering tasks, drivers can allocate the control authority with regard to the haptic guidance system by adjusting the arm admittance [4]. Previous research has suggested a relationship between arm admittance and grip strength on the steering wheel [26] as measured by sEMG signals from forearm muscles [27]. Inspired by research on the grip-force-based scheduling of guidance forces [28], our previous study adjusted the authority of shared steering control according to driver grip strength on the steering wheel to achieve better driver–automation cooperation performance [29]. We found a reduction in both lane departure risk and driver workload to be associated with sEMG-based adaptive authority compared to fixed authority [29].

However, the effect of haptic guidance with adaptive authority on distracted driver behavior remains unknown. As driver distraction is a well-known cause of traffic collisions worldwide, the effectiveness of the proposed shared steering control remains to be tested. Although adaptive automation relieves the driver of the task of engaging and disengaging the automation, it imposes an additional task of monitoring the time-varying automation level of the adaptive system with the possibility of increased workload [22]. Moreover, several experimental studies have indicated that the steering effort of drivers may be even higher for haptic guidance than for manual driving [4,5]. This situation may be more complicated when drivers become distracted. Furthermore, humans have a good ability for adaptation to take advantage of the shared steering control system. When the drivers are under a distracted state due to the secondary task, they may change their adaptive behavior.

Therefore, this study aimed to investigate the effect of sEMG-based adaptive shared control on distracted driver behavior by comparing with an attentive driver state. Fixed shared steering control and manual driving are also addressed for comparisons. It is hypothesized that the adaptive haptic guidance would provide more driving safety and comfort to distracted drivers, whereas fixed haptic guidance could more effectively reduce the duration of a lane change. This study is expected to especially benefit distracted drivers by contributing to safer and smoother steering performance in lane changing as well as lane following tasks under haptic shared control.

## 2. Materials and Methods

### 2.1. Participants

Eighteen healthy subjects (two women and 16 men) were recruited to participate in the experiment. Their ages ranged from 21 to 32 years (mean = 23.5 years, SD = 2.8 years). All subjects had a valid Japanese driver’s license with driving experience (mean = 2.7 years, SD = 2.6 years). The experiment was approved by the Office for Life Science Research Ethics and Safety, Graduate School of Interdisciplinary Information Studies, University of Tokyo (No. 12 in 2017). Each subject provided written consent to the experimental protocol and received compensation for their participation.

### 2.2. Apparatus

**Figure 1:**
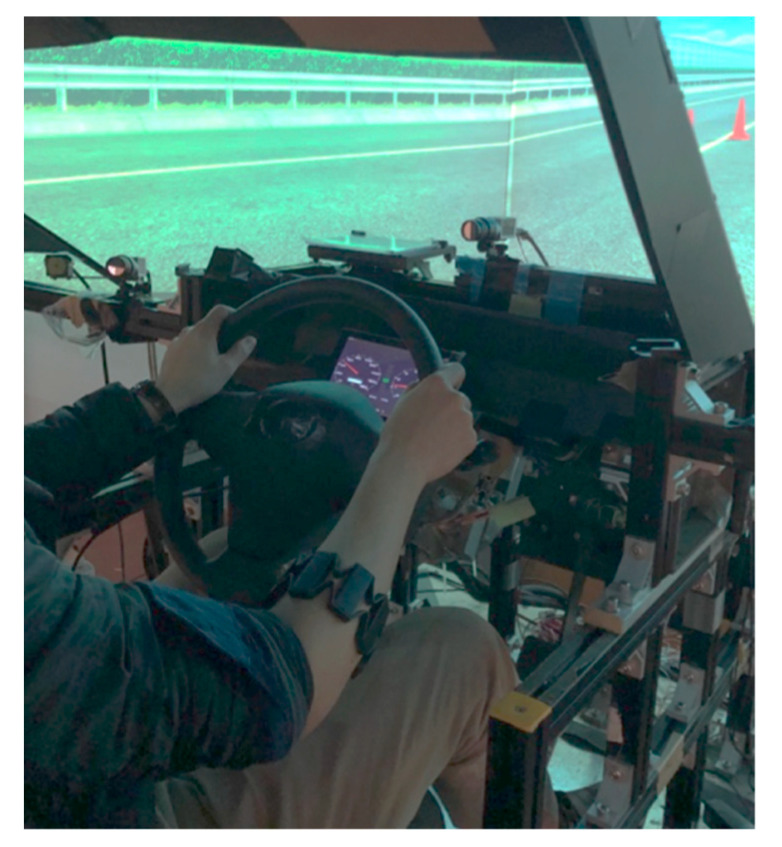
Active steering system and armband worn by driver to enable sEMG-based operation of a high-fidelity driving simulator.

A Myo armband (Thalmic Labs, Inc., Waterloo, Canada) acquired the sEMG from the dominant forearm of the driver (Figure 1). The sEMG was conditioned for further processing by calculating the root mean square (RMS) value of the activation signal from the stainless-steel armband sensors [30]. Driver grip strength was normalized with respect to the maximum sEMG for each participant (sEMG_REF_). The steering wheel torque provided by the adaptive haptic guidance was real-time computed based on the normalized sEMG value.

The experiment was conducted in a moving-based driving simulator with brake and accelerator pedals, an actuated steering wheel, and an instrument dashboard. The driving simulator included a 140° field-of-view and the moving platform had six degrees of freedom, which was considered to be high-fidelity. In order to emulate the feeling of on-road driving, high-frequency vibrations were also produced by the moving platform, engine sounds were provided by two speakers, and a self-aligning torque was generated by the actuated steering wheel. The electronic steering system was connected to the host computer of the driving simulator through a controller area network. The electronic steering system comprised a steering wheel, servo motor, and electronic control unit (ECU). The real-time haptic guidance torque calculated by the host computer served as the input to an ECU. A servomotor was subsequently actuated by the ECU to apply a haptic guidance torque to the steering wheel. The haptic guidance torque was calculated based on a model with two look-ahead points, whereas the magnitude and direction of the haptic guidance torque were determined by comparing the target vehicle trajectory based on the fifth-degree Bezier curve with the actual trajectory of the simulated vehicle [29].

### 2.3. Experimental Conditions and Scenario

The participants drove under six conditions as shown in Table 1. Two driver states were considered: attentive and distracted. For each state, there were three types of haptic guidance: manual steering or HG-Non, HG-Fixed (haptic guidance with fixed authority), and HG-Adaptive (haptic guidance with adaptive authority). Using a 6 × 6 Latin square, the sequence in which the experimental conditions were presented to the participants was partially counterbalanced. The three Latin squares partially counterbalanced the within-subject order of the conditions.

To induce driver distraction, a challenging secondary task, called the paced auditory serial addition task, was applied during the entire driving course. The subjects were given a number every 3 s and were asked to add the number they just heard with the number they heard before. The attentive experimental condition was a control session in which the driver was under normal driving conditions.

For manual driving, the overall gain of haptic guidance was set to 0, whereas the overall gain of the haptic guidance torque was held at 0.25 for HG-Fixed. The normalized torque was based on 25% of the gain for an automated double lane change (DLC). To manage the driver–automation conflict, the sEMG-based adaptive shared steering control was designed to reduce the authority of haptic guidance when the driver increased the grip strength to gain more manual control authority and vice versa. Specifically, the gain of the haptic guidance torque was computed in real time, and it decreased linearly from 1 (gain for automated DLC) to 0, when the driver’s grip strength increased from 0 to sEMG_REF_. Grip strength above sEMG_REF_ during the DLC task prompted adjustment of the haptic guidance gain to 0.

**Figure 2:**
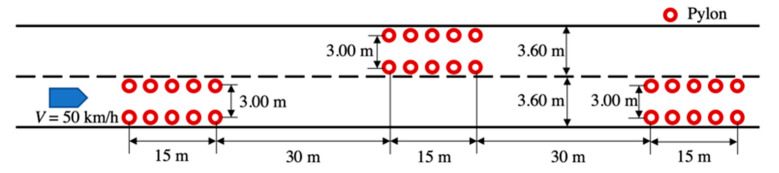
Experimental driving scenario performed in the driving simulator.

The DLC task was composed of two stages of lane changing as illustrated with pylons in Figure 2. Before the DLC section, there was a straight lane 300 m long and, thus, the driver was asked to perform a lane keeping task. The speed was controlled by the driving simulator, and the driver did not need to operate the gas pedal. The initial speed was set at 0 km/h, and the acceleration rate was 1.8 m/s^2^. After reaching 50 km/h at approximately 50 m from the starting point, the driving speed was kept constant. The aim was to motivate the driver to adapt their steering behavior, especially for driving with the haptic guidance. In order to determine the speed value, we conducted a pilot test to compare speeds of 40, 50, and 60 km/h given the same DLC task shown in Figure 2. According to the lane change performance evaluation, the speed of 40 km/h was found to be less challenging, whereas 60 km/h was overly challenging. Therefore, to improve the replicability of the experimental results with respect to steering behavior, a PID controller automatically maintained the simulated vehicle speed at 50 km/h.

### 2.4. Experimental Procedure

Considering that different drivers have different usage habits with their left and right hands, each participant was asked about his/her dominant hand so that the armband was mounted on the dominant forearm. The sEMG normalization was realized by having each participant grip the driving simulator steering wheel in a “ten-and-two” position for 2 s with maximum grip strength. This procedure was repeated thrice with 10 s of rest between each repetition. The mean value across all repetitions for a given participant was used as the reference sEMG value, sEMG_REF_, for normalization.

Each subject performed a practice task prior to the actual experiment so that they were accustomed to operating the simulator. Throughout the practice and experimental trials, drivers were trained to maintain both hands at the ten-and-two steering wheel position. The drivers repeated the DLC task five times for each of the six driving conditions and, thus, each driver performed a total of 30 trials for the actual experiment. The subjective task load was measured by having each participant complete a questionnaire after each trial.

### 2.5. Measured Variables

The DLC performance was evaluated based on driver steering behavior, lane departure risk, and subjective evaluation. The measured driver input torque, DLC duration, steering wheel angle (SWA), lateral acceleration, and normalized sEMG constituted the evaluation of driver steering behavior. The normalized sEMG RMS (sEMG/sEMG_REF_) was calculated from the measured forearm signals. An increased normalized value indicated greater grip strength. The driver steering effort was determined by the calculated RMS value of the driver input torque. Calculating the RMS value of the SWA and peak SWA determined the magnitude of the steering control activity. The DLC process consisted of two parts: the first lane change part and the second lane change part. The peak value of SWA for each lane change part was calculated. The duration of DLC was calculated to express the timing of lane change maneuvers in a quantitative way. The starting point of the DLC was determined when the vehicle yaw angle was higher than 1.0 degree through a trial-and-error process. The duration of DLC was calculated from the starting point to the ending point. The driver normally made a small adjustment at the ending point to return to the centerline of the lane. Hence, the yaw rate was considered with yaw angle to determine the ending point. Furthermore, the ending point of the DLC was determined by trial-and-error process to correspond to a yaw angle and yaw rate both below 0.5 degrees.

At the conclusion of each lane change stage with the simulated vehicle driving parallel to the entered lane, the lateral error relative to the centerline of the lane was measured. The lane departure risk during DLC was evaluated using the lateral error.

Subjective preferences for the different types of tested haptic guidance paired with each driver state were recorded in conjunction with the NASA task load index (NASA-TLX) to conduct a subjective evaluation. The participants rated their workload according to the index after each driving condition. At the conclusion of all experimental trials, each participant selected the experimental condition with the highest degree of satisfaction. The preference score for a given condition equaled the number of times the condition was selected across all participants. Dividing the preference score by two yielded a relative preference score.

### 2.6. Analysis

In accordance with two-way repeated-measures analysis of variance (ANOVA), the extent of interaction between the state of the driver and haptic guidance that affected driver behavior was determined. Setting the level of significance to *p* = 0.05, Mauchly’s test was executed before the repeated-measures ANOVA. Furthermore, Fisher’s least significant difference for pairwise comparisons identified the main effects with a selected significance criterion of *p =* 0.05. Differences were considered statistically significant when the *p*-value ≤ 0.05, and a *p*-value ≤ 0.1 was interpreted as a tendency toward statistical significance.

## 3. Results and Discussion

The results in this section are described with regard to driver steering behavior and lane departure risk in addition to subjective evaluation. The *p*-values for two-way repeated-measures ANOVA related to driver behavior as well as the corresponding mean and standard deviations for experimental variables are listed in Table 2. This includes the main effect for the driver state, main effect for HG, and interaction effect between the drive state and HG.

The main effect was significant for HG in terms of the RMS of driver input torque (*p* < 0.001), lateral error at the end of the first lane change (*p* < 0.001), and relative score of pairwise preference (*p* < 0.01), although there was no significance for the main effect of driver state and interaction effect. As for the peak value of SWA in the first lane change, the main effect was significant for HG (*p* < 0.05) and driver state (*p* < 0.05). As for the peak value of SWA in the second lane change, there was a tendency toward significance for the HG (*p* < 0.1) and driver state (*p* < 0.1). The results of peak value for lateral acceleration had a similar tendency as the peak value of SWA. As for DLC duration, the main effect was significant for HG (*p* < 0.01), and there was a tendency toward significance for the interaction effect (*p* < 0.1). The main effect was significant for the driver state in terms of lateral error at the end of second lane change (*p* < 0.05) and overall workload according to the NASA-TLX (*p* < 0.001), whereas no significant difference was observed for the main effect of HG and the interaction effect.

### 3.1. Driver Steering Behavior

The results of driver input torque are shown in Table 2. From pairwise comparisons, the driver input torque for manual was significantly greater than that for HG-Fixed (*p* < 0.001), greater for manual than for HG-Adaptive (*p* < 0.001), and greater for HG-Fixed than for HG-Adaptive (*p* < 0.001). Therefore, our hypothesis was validated because haptic guidance significantly reduced driver steering effort, and HG-Adaptive was more effective.

**Figure 3:**
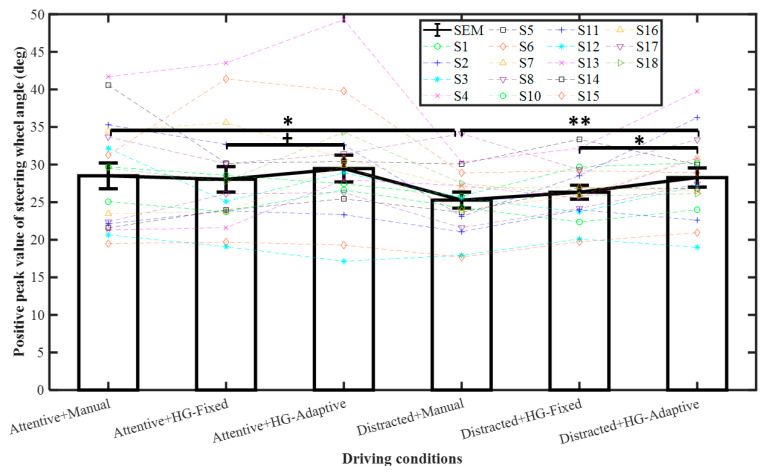
Peak value of SWA at the first lane change. Mean +/− SEM (standard error of mean) represented by error bars. ^+^ *p* < 0.1, * *p* < 0.05, and ** *p* < 0.01.

The results of peak value for SWA in the first lane change are shown in Table 2 and Figure 3. The steering wheel angle and lateral acceleration data from Subject no. 9 were eliminated due to the fact of its extreme deviation from the data of other subjects when plotting the figure and conducting the statistical analysis. For the distracted state, the peak value of SWA was significantly lower for manual steering than for HG-Adaptive (*p* < 0.01) and lower for HG-Fixed than for HG-Adaptive (*p* < 0.05). Moreover, the peak value of SWA for manual steering was significantly higher with the attentive state than with the distracted state (*p* < 0.05). Hence, the distraction reduced the steering activity, and the haptic guidance system increased the steering activity to a level comparable to that of the attentive state. A similar tendency was found for the RMS of the SWA, although the difference was not statistically significant for HG. This may be due to the effect of HG and distracted state on SWA being less pronounced during the second lane change compared with first lane change as shown in Table 2. Thus, a more demanding secondary task or lane change task will be considered in future studies. Moreover, we found that there was a high correlation and similar tendency between the results of steering wheel angle and lateral acceleration. In this study, lateral error was mainly used to evaluate the lane departure risk. If there was no significant difference for lateral error, a lower lateral acceleration would indicate a smoother lane change with less steering control activity, thus implying a relatively lower risk of lane departure.

**Figure 4:**
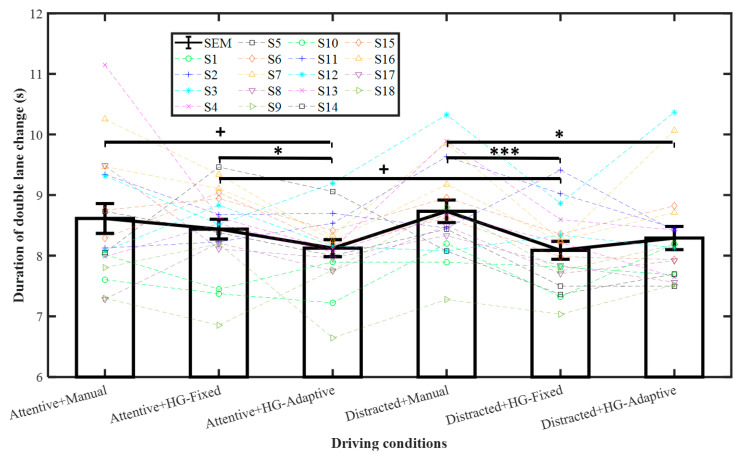
Double lane change duration. Mean +/− SEM represented by error bars. ^+^ *p* < 0.1, * *p* < 0.05, and *** *p* < 0.001.

The results for the DLC duration are shown in Table 2 and Figure 4. From pairwise comparisons, for attentive, the duration of the DLC was significantly shorter with HG-Adaptive than with HG-Fixed with *p* < 0.05, whereas the DLC tended to be significantly shorter for HG-Adaptive than for manual steering, where *p* < 0.1. For distracted, the DLC duration was significantly shorter with HG-Fixed than with manual (*p* < 0.001) and shorter with HG-Adaptive than with manual (*p* < 0.01). Moreover, for HG-Fixed, there was a tendency for the DLC duration to be shorter with distracted than with attentive (*p* < 0.1). Therefore, HG-Fixed could more effectively reduce lane change duration for distracted drivers as predicted by our hypothesis.

**Figure 5:**
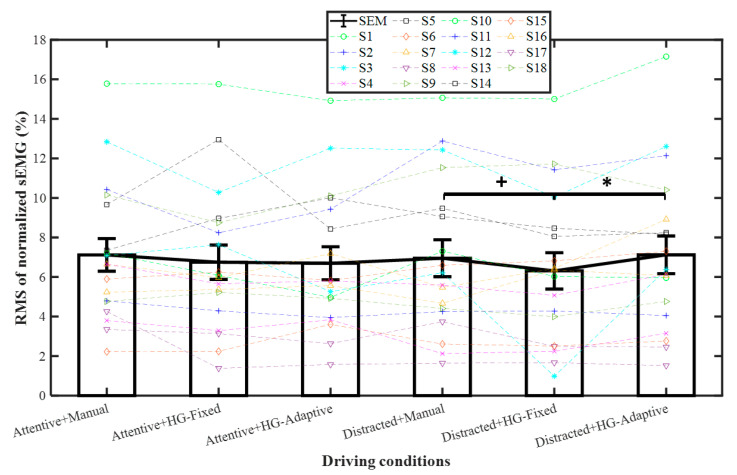
RMS of normalized sEMG (%). Mean +/− SEM represented by error bars. ^+^ *p* < 0.1 and * *p* < 0.05.

The RMS results of sEMG are shown in Table 2 and Figure 5. Based on pairwise comparisons, for distracted, the RMS of sEMG was significantly lower with HG-Fixed than with HG-Adaptive (*p* < 0.05) and lower with HG-Fixed than with manual (*p* < 0.1), indicating that distracted drivers tended to give more control authority to the HG-Fixed by reducing grip strength. Consequently, the DLC duration was relatively shorter (Figure 4).

### 3.2. Lane Departure Risk

**Figure 6:**
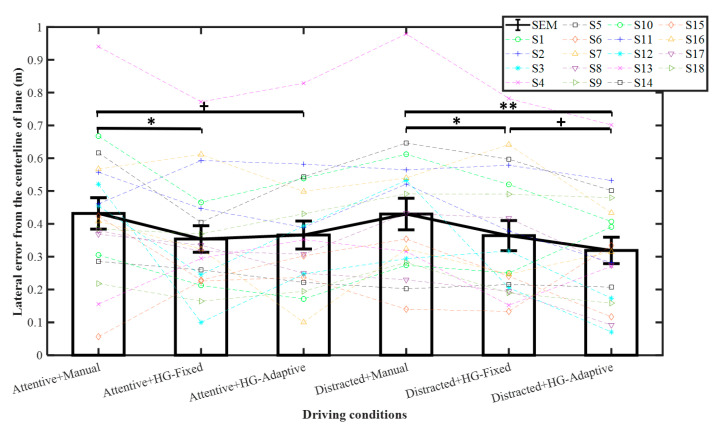
Lateral error with respected to lane centerline at end of first lane change. Mean +/− SEM represented by error bars. ^+^ *p* < 0.1, * *p* < 0.05, and ** *p* < 0.01.

Figure 6 and Table 2 show the results of the lateral error at the end of the first lane change. Pairwise comparisons indicated that for the attentive condition, the lateral error was significantly higher for manual steering than for HG-Fixed with *p* < 0.05 and tended to be higher for HG-Adaptive with *p* < 0.1. The lateral error for distracted was significantly lower in the case of HG-Fixed than in manual, where *p* < 0.05; HG-Adaptive was significantly lower than HG-Fixed (*p* < 0.01). Furthermore, HG-Adaptive tended to be significantly lower than HG-Fixed with *p* < 0.1. Thus, haptic guidance can reduce lane departure risk when the driver is attentive as confirmed by our previous study [29]. This outcome could be attributed to the human driver being limited by the response of the neuromuscular system, thereby making it difficult to complete the DLC accurately [31]. Furthermore, from this result, haptic guidance is also capable of reducing the lateral error for distracted drivers with HG-Adaptive being more effective.

The results for lateral error in the case of the second lane change are listed in Table 2. The lateral error was significantly increased by driver distraction, but the effect of haptic guidance was insignificant. According to the peak SWA in the second lane change, no significance was observed with regard to the effect of HG. In accordance with our previous study [29], the lateral error at the end of first lane change was almost twice as large than at the end of the second lane change. The proposed haptic guidance system was more effective for the first lane change than the second one. This could be because there was more frequent overshoot during the first lane change, and haptic guidance was effective in diminishing the overshoot. Moreover, HG-Adaptive was more effective than HG-Fixed when the driver was distracted.

### 3.3. Subjective Evaluation

**Figure 7:**
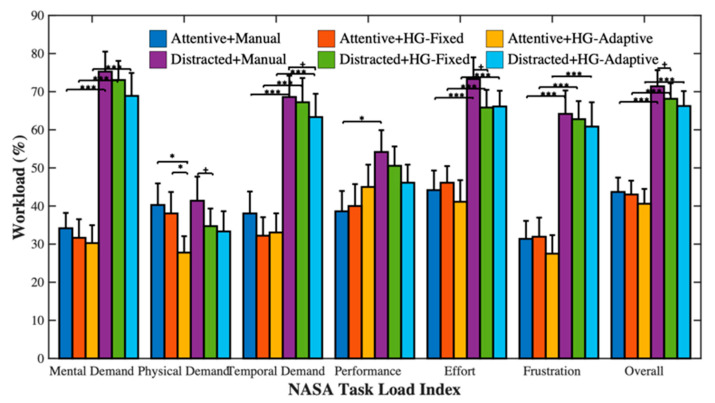
Mean scores on NASA-TLX. Data error bars represent the mean + SEM. ^+^ *p* < 0.1, * *p* < 0.05, and *** *p* < 0.001.

The results of the driver workload assessed by the NASA-TLX are shown in Table 2 and Figure 7. Taking into account the pairwise comparisons, the overall driver workload for distracted was significantly higher than for attentive (*p* < 0.001). Moreover, for distracted, HG-Fixed yielded a lower overall workload (*p* < 0.1), lower physical demand (*p* < 0.1), and lower effort (*p* < 0.1), and HG-Adaptive yielded a lower temporal demand (*p* < 0.1). This indicates that the driver workload increased during the secondary task, and the haptic guidance system effectively reduced the driver workload.

Table 2 shows the relative scores of pairwise driver preferences. For both attentive and distracted, drivers preferred HG-Fixed over manual, HG-Adaptive over manual, and HG-Adaptive over HG-Fixed. Moreover, there was a tendency for more drivers to prefer haptic guidance over the manual when they were distracted, which was expected, as haptic guidance reduces lane departure risk as well as driver workload.

### 3.4. Limitations and Future Works

One limitation of this study is that only a constant gain of 0.25 was considered for the HG-Fixed and linear law (i.e., linearly decreasing from 1 to 0 as the driver grip strength increased from 0 to sEMG_REF_) for the HG-Adaptive. In the current study, the linear law was employed because it was assumed to be more intuitive to the drivers and could act as a benchmark and a reference for subsequent studies. Considering that drivers might not have the same behaviors when the fixed gain or adaptive strategies are changed, future studies could address other fixed gains and other laws (e.g., sigmoid law) for the haptic guidance design. Another limitation of the current work is that the driver distraction state was not measured. Considering that driver distraction could be estimated by monitoring eye and head movements in real time, an adaptive shared steering control based on multimodal sensory signal processing will be designed in our future work. Moreover, the driving speed was kept constant in the current study, resulting in less natural driving behavior. In the future study, more natural driving conditions with speed variability and a haptic shared controller design that takes into account speed variation will therefore be addressed. The current study only focused on lane change behavior on a relatively short driving course. A longer driving scenario with curved roads will be designed as future work, and the sEMG-based adaptive shared steering control system will be investigated for both lane change and lane following tasks. Given that the experimental results in the current study were based on driving simulations, future studies could consider evaluation of sense of presence in relation to virtual environments.

## 4. Conclusions

This driving simulator study focused on the effect of haptic guidance with adaptive authority on distracted driver behavior. DLC tasks were completed according to experimental conditions that were designed by combining two driver states, namely, attentive and distracted, along with three haptic guidance categories: HG-Fixed, HG-Adaptive, and Manual. HG-Adaptive relied on feedback from the real-time forearm sEMG of the driver.

For both attentive and distracted drivers, HG-Adaptive yielded a greater reduction in the driver workload and lane departure risk than that of HG-Fixed and manual steering. Moreover, drivers tended to reduce the steering wheel grip strength to provide admittance to the haptic guidance with fixed authority, resulting in the completion of the DLC in a relatively short period. As the current study indicated a small lane departure risk induced by driver distraction, we plan to address the possibility of greater risk by conducting a future study with a more demanding secondary task or lane change task. Although the current experiment was conducted in a high-fidelity driving simulator, future work will address how driver behavior would be in a more natural driving setting via real-vehicle experiments. Furthermore, the sample size of the present study was relatively small and biased toward male drivers and, thus, data from more female drivers should be collected in future work to further assess the effectiveness of the proposed system.

## Figures and Tables

**Table 1:** Experimental conditions.

Condition	Driver State	Haptic Guidance
1	Attentive	Manual (HG-Non)
2	Attentive	HG-Fixed
3	Attentive	HG-Adaptive
4	Distracted	Manual (HG-Non)
5	Distracted	HG-Fixed
6	Distracted	HG-Adaptive

**Table 2:** Experimental conditions.

Variable	Attentive and Manual (1) M (SD)	Attentive and HG-Fixed (2) M (SD)	Attentive and HG-Adaptive (3) M (SD)	Distracted and Manual (4) M (SD)	Distracted and HG-Fixed (5) M (SD)	Distracted and HG-Adaptive (6) M (SD)	Driver State *p*-Value	HG *p*-Value	Interaction *p*-Value
RMS of driver input torque (N·m)	1.029 (0.064)	0.734 (0.072)	0.633 (0.106)	1.029 (0.058)	0.722 (0.081)	0.643 (0.149)	0.941	0.000 ***	0.454
RMS of SWA (degree)	18.897 (3.182)	19.122 (2.551)	19.403 (2.674)	17.976 (2.015)	18.592 (2.058)	18.555 (2.049)	0.059 ^+^	0.198	0.732
Peak value of SWA at the 1st LC (degree)	28.497 (7.116)	28.040 (6.986)	29.468 (7.394)	25.274 (4.366)	26.323 (3.826)	28.274 (5.265)	0.046 *	0.024 *	0.203
Peak value of SWA at the 2nd LC (degree)	−33.815 (6.301)	−33.135 (5.184)	−32.867 (5.763)	−33.035 (4.758)	−31.296 (3.052)	−31.203 (3.811)	0.056 ^+^	0.092 ^+^	0.595
Peak value of lateral acceleration at the 1st LC (m/s^2^)	1.702 (0.437)	1.670 (0.418)	1.761 (0.445)	1.501 (0.264)	1.571 (0.229)	1.684 (0.313)	0.046 *	0.026 *	0.180
Peak value of lateral acceleration at the 2nd LC (m/s^2^)	−1.983 (0.381)	−1.944 (0.312)	−1.936 (0.356)	−1.929 (0.280)	−1.828 (0.189)	−1.833 (0.228)	0.047 *	0.147	0.619
Duration of double LC (s)	8.613 (1.044)	8.437 (0.689)	8.123 (0.595)	8.731 (0.788)	8.088 (0.626)	8.290 (0.811)	0.867	0.002 **	0.083 ^+^
RMS of normalized sEMG (%)	7.116 (3.501)	6.746 (3.686)	6.696 (3.547)	6.947 (3.966)	6.309 (3.908)	7.120 (4.053)	0.822	0.099 ^+^	0.208
Lateral error at the end of 1st LC (m)	0.432 (0.203)	0.354 (0.172)	0.366 (0.181)	0.430 (0.204)	0.364 (0.195)	0.319 (0.171)	0.391	0.000 ***	0.296
Lateral error at the end of 2nd LC (m)	0.193 (0.111)	0.214 (0.123)	0.169 (0.100)	0.241 (0.099)	0.215 (0.101)	0.224 (0.133)	0.033 *	0.650	0.114
NASA-TLX overall workload	43.685 (16.020)	43.019 (15.408)	40.593 (16.489)	71.407 (17.924)	68.130 (17.330)	66.241 (16.541)	0.000 ***	0.166	0.826
Relative score of pairwise preference	0.647 (0.786)	0.882 (0.781)	1.471 (0.624)	0.353 (0.702)	1.059 (0.748)	1.588 (0.507)	1	0.001 **	0.314

^+^ *p* < 0.1, * *p* < 0.05, ** *p* < 0.01, and *** *p* < 0.001. SWA: steering wheel angle; LC: lane change; HG: haptic guidance.

## Data Availability

The data for this study are available in the IEEE DataPort: 10.21227/ckxa-8655 (accessed on 12 May 2021).
